# Environmental monitoring of linear alkylbenzene sulfonates and physicochemical characteristics of seawater in El-Mex Bay (Alexandria, Egypt)

**DOI:** 10.1007/s10661-012-2776-9

**Published:** 2012-08-01

**Authors:** M. A. Okbah, A. M. A. Ibrahim, M. N. M. Gamal

**Affiliations:** 1 National Institute of Oceanography and Fisheries, Alexandria, Egypt; 2 Faculty of Science, Alexandria University, Alexandria, Egypt

**Keywords:** Anionic detergents, LAS, Seawater, Physicochemical, El-Mex Bay

## Abstract

In the present work, the influence of different physicochemical characteristics on the distribution of anionic detergents, linear alkylbenzene sulfonates (LAS), was studied. Surface and bottom water samples were collected from eight different sites from a small bay near the main sewage discharge of Alexandria City (El-Max Bay). The results showed great variations in the concentrations, as a function of the regional and seasonal variations. The study revealed that the pH values lie in the normal side, with a range of 8.0–8.5 inside the bay and 7.5–7.7 at El-Umum Drain effluent. Wide variations, observed between the surface and the bottom water of the bay, salinity, dissolved oxygen, oxidizable organic matter, total hardness, and total alkalinity, were scattered in the ranges (3.33–42.73 practical salinity unit), (0.42–8.27 mg O_2_/l), (0.12–10.49 mg/l), (1.39–8.99 mg/l), and (0.23–0.48 mg/l), respectively. The regional variations of LAS concentrations in the bay waters showed that the concentration decreased as the distance from the source of drainage water (El-Umum Drain). The seasonal average variations of LAS cleared out that summer and spring periods had the highest concentrations at surface (0.13 ± 0.04 mg LAS/l) and bottom (0.12 ± 0.10 mg LAS/l) layer, which is attributed to increase in population density and human activities. The inverse relationships between total LAS concentration and salinity, dissolved oxygen, and calcium ions concentration are *r* = −0.78, 0.50, and 0.67, respectively. This is related to the occurrence of the untreated wastewater containing detergents, the biodegradation rate of surfactants, and strong precipitation of LAS as Ca.

## Introduction

El-Mex district is an industrial zone, west of Alexandria City. As a consequence of growing heavy industries (petrochemicals, pulp metal planting, industrial dyes, and textiles) and the uncontrolled disposal of the resulting waste, the costal water of El-Mex Bay receives huge amounts of untreated industrial wastes (Shriadah and Emara 1996; Fahmy et al. 1997). These wastes are containing potentially toxic materials, which are pumped directly into the bay via a pipeline in its southern part. El-Mex Bay receives a heavy load of wastewater (2.4 × 10^9^ m^3^/year) both directly to the sea from industrial outfalls and indirectly from Lake Maryout via El-Mex (Said et al. 1994). Pumping station, which lies about 1 km upstream on El-Umum Drain, is mainly agricultural drainage water collected by El-Umum Drain but also comprises the overflow from Lake Maryout (Masoud et al. 2001). Close proximity of a cement factory contributes significant amounts of cement dust to the bay water. Shipping activities along the main trade harbor (Western Harbor) contribute to bay pollution (Samir and El-Din 2001). For these reasons El-Mex Bay represents a good model for the environmental exposure for different types of pollutants. Chemical substances have a considerably negative impact on the environment during all their life stages (Schröder et al. 1999). The exact percentage of the chemical released into the environment mainly depends on the physicochemical and biological properties of the chemical as well as the way it is used and disposed of linear alkylbenzene sulfonates (LAS) as anionic surfactants and used in detergents and cleaners as intermediates in chemical synthesis. These chemicals show a pronounced ecotoxicological effect on aquatic organisms and used in large quantities as consumer products which are discharged into wastewater after usage. The harmful effects of detergents in natural waters may also results in a general impact on the biogeochemical cycle of other pollutants and biogenic elements. Solubility of heavy metals and mineral oil components is increased in the presence of detergents. Monitoring and treatment of the sewage is the key for protecting the environment from the negative impact of these chemicals.

The objective of the present study is to investigate the hydrographic conditions of El-Mex Bay water and their influence on the distribution and seasonal variations of linear alkylbenzene sulfonates in the bay water.

## Material and methods

### Area of study

**Fig. 1:**
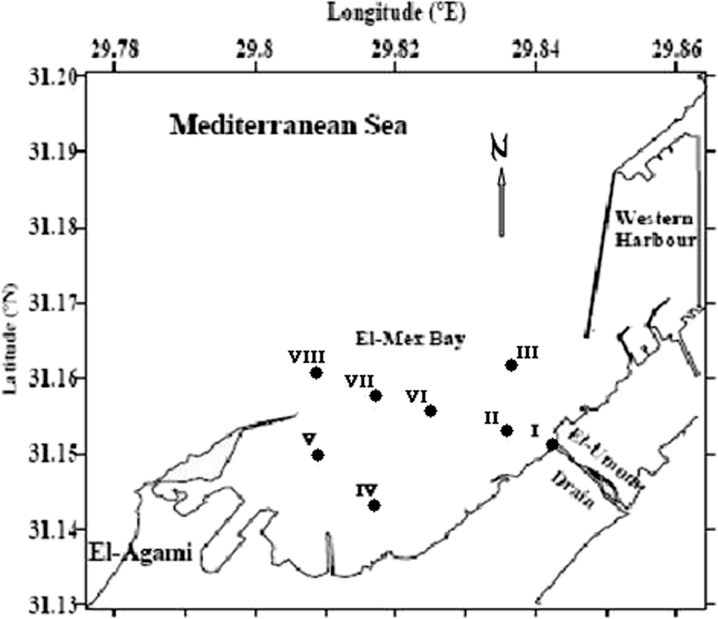
Sampling location

El-Mex Bay lies in the west of Alexandria at longitude 29°78′ E and latitude 31°13′ N, and it extends for about 15 km between El-Agamy headland to the west and the Western Harbor to the east and from the shoreline seaward to a depth of 20 m (Fig. 1). Its surface area is about 19.4 km^2^, and its volume 190 × 10^6^ m^3^. In general, the shoreline is rocky with narrow sandy beaches surrounding the embayment. There are pronounced differences in both the direction and intensity of marine currents in the bay near the outlets (Samir and El-Din 2001). The bay is also characterized by the presence of an eddy current affecting most of its parts.

### Sampling and analysis

Sixteen surface and bottom water samples were collected from El-Mex Bay, using Niskin water sampler, during 2008 (Fig. 1) using a motor boat. The sampling program provided seasonal basic measurements of the physicochemical parameters of the bay waters. Measurements were always carried out in the same order during the sampling day in order to keep to a minimum the fluctuations of the physical and chemical parameters caused by temperature differences. Water samples were collected in plastic vessels and shipped to the laboratory the same day of samples collection. Upon arrival, the samples were preserved in a refrigerator at 4 °C prior to analysis

Hydrographic and some chemical parameters were measured in situ; water temperature and pH were measured directly in the field using a standard Schmidt thermometer and a portable digital pH meter (Orion Research, model 210), respectively. Salinity was determined using a Beckman induction salinometer (model RS-7 C). The classical Winkler method was used for the determination of dissolved oxygen (Grasshoff 1983). Determination of total alkalinity was carried out according to Strickland and Parsons (1972).

The method used for the determination of oxidizable organic matter (OOM) is described by FAO (1975). A water sample of 100 ml was boiled with a mixture of 10 ml alkaline 0.01 N KMnO_4_ solution and one pellet of NaOH for 20 min and then cooled to room temperature. Five milliliters of 9 N H_2_SO_4_ were added to the previous mixture followed by adding 0.5 g KI. The librated iodine was then titrated against standard of 0.02 N sodium thiosulphate solution using starch as indicator. During titration, a yellow color appeared and then turned to blue in the presence of the solution, and finally, the endpoint of titration turned the mixture to colorless. Blank experiment was made by treating of 100 ml distilled water in the same way explained above. The preferred method for determining total hardness is ethylene diaminetetraacetic acid titrimetric method described by APHA (2005) for the examination of water and wastewater.

### Analysis of LAS in seawater

Rapid determination of surfactants (LAS) by spectrophotometer method using methylene blue (MB), as cationic dye, was investigated by Koga et al. (1999). The method was based on the reaction between linear alkylbenzene sulfonates, as anionic surfactants, and methylene blue, as cationic dye, to form associated ion pair (LAS-MB ion pair) in water with 1:1 molar ratio which could be easily extracted to the organic phase (chloroform, CHCl_3_). The measurements of LAS were performed using a Shimadzu double-beam spectrophotometer UV-150-02.

### Method validation and quality control

All chemicals were of analytical reagent grade, and sodium dodecylsulfate was employed as a representative anionic surfactant for calibration. It was obtained from Sigma (St. Louis, USA) (SDS [CH_3_(CH_2_)_11_OSO_3_Na]). Methylene blue (trihydrates) was used as a cationic dye and obtained from Merck (Steinheim, Germany) (MB [C_16_H_18_N_3_SCl·_3_H_2_O]). Chloroform stabilized with ethanol (Scharlau, Barcelona, Spain) was used as extractant. For other products, analytical grade reagents were used.

Validation of the method and quality control samples was done using a reference material (Anionic Surfactant–WP Mfr/RTC). The limit of detection was calculated by six determinations (duplicate measurements) in one batch of synthetic seawater. The detection limits was 0.001 mg LAS/l. Precision was determined by three replicate analyses of one sample and expressed as a coefficient of variation, and the result of the precision agreed within 10 %. The accuracy of LAS determination was evaluated by spiking 1 l of synthetic seawater using certified reference material (Anionic Surfactant–WP Mfr/RTC). The spiked samples were extracted using the previous method, and a LAS concentration was determined. The recovery of spiked LAS was 95 %.

## Results and discussion

### Physicochemical characteristics of El-Mex Bay

#### Temperature

**Fig. 2:**
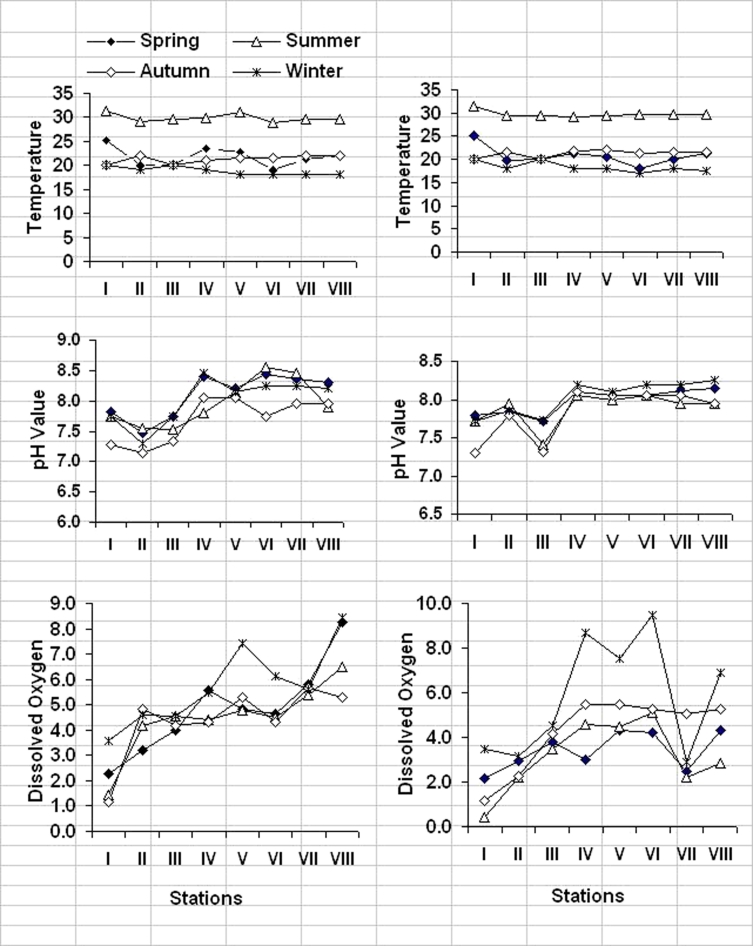
Regional and seasonal variations of temperature (in degrees Celsius), pH values, and dissolved oxygen (in milligrams per liter) in El-Mex Bay Water (2008)

The distribution pattern of water temperature showed that the surface water temperatures were generally higher than those of the bottom as shown in Fig. 2. Surface water temperature varied from a minimum of 18 °C in March to a maximum of 31 °C in August. The bottom water temperature is relatively higher than that of the surface water at some stations where the samples collection was performed during the summer season (August), while it is relatively lower throughout the year, with a difference of 0.5–2.3 °C. This indicates that the variation in bay water as a results of seasonal variations of air temperature and solar radiation.

#### Salinity

**Fig. 3:**
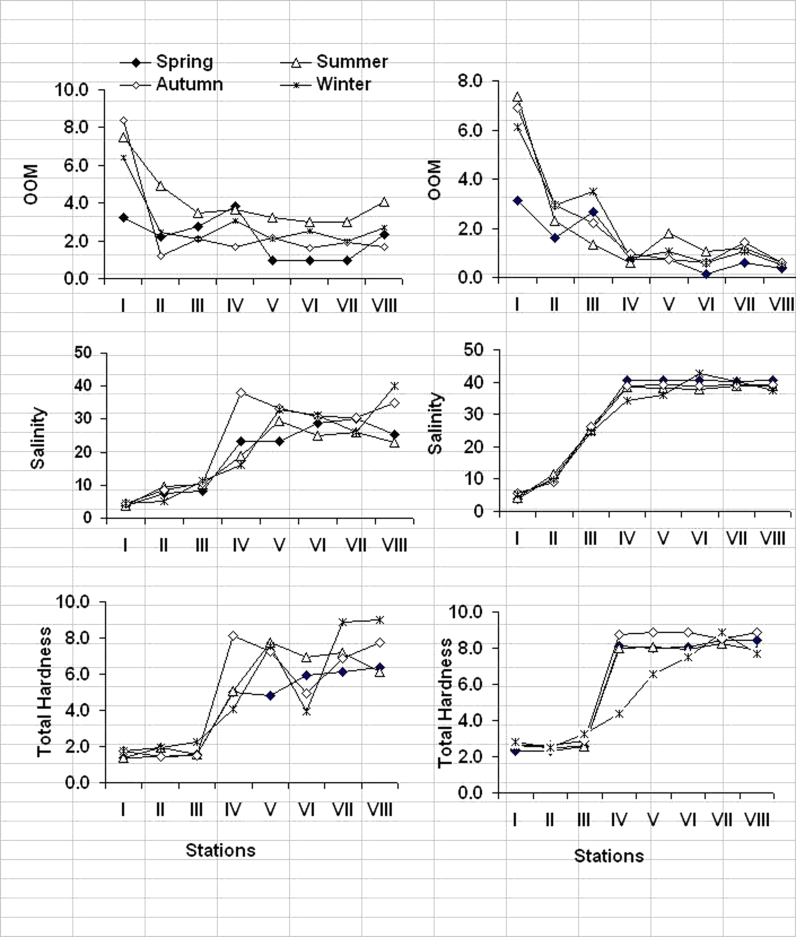
Regional and seasonal variations of oxidizable organic matter (in milligrams per liter), salinity (in practical salinity units), and total hardness (in milligrams per liter) in El-Mex Bay Water (2008)

In present study, salinity is used as indicator to reflect changes resulting from the mixing of fresh and seawaters. Salinity of El-Mex Bay was mostly affected by the amount of drainage water of El-Umum drain and the rate of exchange with the adjoining open sea. The regional and seasonal surface and bottom salinities and the corresponding water types are represented in Fig. 3. Salinity values showed a big variation according to the distance of the different sites from the effluents. The minimum surface salinity (3.71 practical salinity unit (PSU)) was observed during summer season within El-Umum Drain at station I and the maximum (40.09 PSU) found in spring season (March) at the open sea stations. Vertically, salinity distribution in the bay showed a noticeable increased in the bottom waters (>38.10 PSU), compared to those in the surface water (4.0–31.0 PSU). Such high subsurface salinity values can be related to the lower current flow from the open sea to the investigated region (Said et al. 1994; Tayel et al. 1996).

Based on the distribution of surface salinity in the investigated area, four types of water are identified (Said et al. 1994): Mediterranean seawater (S) of salinity > 38.50 PSU, diluted seawater (D) with a salinity range from 30.00 to 38.50 PSU, mixed water (M) salinity 10.00 to 30.00 PSU, and mixed land drainage (L) with a salinity of less than 10.00 PSU. The salinity value 38.50 PSU was taken to represent the inner boundary of the neritic water off Alexandria. This value, mentioned here, is used to identify the limits within which the diluted seawater extends horizontally seawards.

The surface salinity during the winter season revealed mixing water type (salinity ranged from 10 to 30 PSU). Salinity more than 38.50 PSU could only be found at the northern part of the investigated area, as a result of the increased of human activities and the consequent increased in the discharged drainage water in summer seasons.

#### pH

pH values play an important role in many life processes in the sea. It may also reflect the productivity and pollution levels of the aquatic environments. pH values were lower at the shallow stations (pH of natural seawater is around 8.2), and it ranged between 7.5 and 7.7. The lower pH values were observed at stations I (inside El-Umum Drain) and III (near El-Umum Drain). This explains the effects of different effluents in the surface water of El-Mex Bay. Moreover, the vertical pH values in the bay showed relatively high levels in the surface waters, comparing with those recorded in the bottom waters. This could be explained by the regional pH values (Fig. 2); whereas the spatial variation of pH values in the bay was limited within the stations during the year, it ranged between 8.09–8.25 for surface layers and 8.05–8.11 for bottom layers.

Thus, an increase in the phytoplankton population produces an increase in the pH value and oxygen super saturation due to high photosynthetic activity (Sawidis and Bellos 2005). This evidence was supported by the positive correlation found in the water pH values with dissolved oxygen (*r* = 0.397, *n* = 48, *p* < 0.005), where the two parameters were used as good indicator for the production level. Seasonal variation of pH values (Fig. 2) did not show great differences, but the relative high values of pH were recorded in warm periods (summer and spring) in the surface water, which could be attributed to the rising of water temperature as well as the increasing in photosynthetic activity by green and blue green algae (El-Sherif and Mahmoud 1991), which leads to reduce the amount of CO_2_ in water (Masoud et al. 2001).

#### Dissolved oxygen

Dissolved oxygen (DO) is considered as one of the most important and useful parameters for the identification of different water masses. Its distribution reflects to great extent local processes of production and consumption. In El-Mex Bay, the absolute surface and bottom DO values fluctuated between the minimum of 0.45, 0.55, and 1.84 mg O_2_/l at stations I, III, and VIII in summer season, respectively, and the maximum of 8.45, 8.70, and 9.47 mg O_2_/l at stations VIII (surface), IV, and VI (bottom), respectively, in spring. Generally, stations I, II, and III of the bay showed low DO levels, and these stations are located inside and near El-Umum Drain. The regional distribution of DO showed irregular variations (Fig. 2). Based on seasonal variations, the surface DO content was lower at stations IV, VI, V, and VII in winter season and stations IV, VI, V, and II in spring season. Generally, during spring and summer, the DO content of the surface water (4.95 and 2.84 mg O_2_/l, respectively) increased than that recorded in the bottom water for the same seasons (spring, 3.38 mg O_2_/l; summer, 2.40 mg O_2_/l). The high values in surface water may be due to the dissolution of O_2_ from the air to the surface water; while the low values in the bottom water may be attributed to respiration of marine organisms and biochemical transformations of organic matter.

The most important factors, controlling the DO budget, were the quantity and quality of the discharged sewage wastes, the exchange of water with the adjoining open Mediterranean waters, and the high rate of photosynthetic activity of phytoplankton production that produce large amounts of oxygen. These conditions were clearly demonstrated in the bay surface water during the year, when the lowest surface dissolved oxygen content in summer and autumn season was accompanied with the highest surface oxidizable organic matter in the same seasons (Fig. 3). Also, in the summer seasons, the lowest DO coincided with the lowest salinity (Fig. 3) at surface layers.

The effect of temperature on DO concentrations was indicated by the strong inverse correlations found between the two variables (*r* = −0.576, *n* = 48, *p* < 0.002). The decreasing in water temperature leads to an increase in oxygen solubility and decrease in the rate of bacterial decomposition (Masoud et al. 2001).

#### Oxidizable organic matter

High values of oxidizable organic matter indicated that water pollution was present, which was linked to sewage effluents discharged into the area (2.4 × 10^9^ m^3^/year). In the present study, the concentrations of the chemically oxidizable organic matter in El-Mex Bay water were investigated and showed spatial and temporal variations. Vertically, the pattern of organic matter distribution showed a decrease in depth at most stations (Table 1 and Fig. 3). The seasonal variation of OOM (Fig. 3) presented abrupt fluctuations during the year due to the external manipulation of the bay.

**Table 1:** Min, max, and average ± standard deviation of physicochemical parameters and surfactant concentration of El-Max Bay waters

Parameters		Spring	Summer	Autumn	Winter
Temperature (°C)	S	19.20–25.10	28.90–31.45	20.00–22.00	18.00–20.00
		21.81 ± 2.13	29.93 ± 0.95	21.21 ± 0.85	18.82 ± 0.88
	B	18.00–25.10	29.20–31.45	20.00–22.00	17.00–20.00
		20.92 ± 2.35	29.85 ± 0.82	21.17 ± 0.78	18.34 ± 1.14
pH value	S	7.74–8.48	7.52–8.55	7.28–8.15	7.75–8.45
	B	7.71–8.14	7.41–8.05	7.30–8.10	7.72–8.25
D O(mg/l)	S	2.98–8.27	1.45–5.17	1.17–6.83	3.58–8.45
		4.91 ± 2.04	4.36 ± 1.81	4.19 ± 2.11	5.79 ± 2.40
	B	2.14–4.32	1.42–5.08	1.18–5.46	2.90–9.47
		3.37 ± 1.07	3.08 ± 1.68	4.07 ± 1.91	5.90 ± 2.65
OOM (mg/l)	S	0.96–3.84	3.00–7.49	1.20–8.40	2.00–6.38
		2.11 ± 1.18	4.33 ± 1.78	3.53 ± 2.68	3.67 ± 1.78
	B	0.12–3.12	0.60–7.33	0.60–6.89	0.52–6.11
		1.21 ± 1.17	3.32 ± 3.56	2.39 ± 2.47	2.32 ± 2.36
Salinity (PSU)	S	3.89–32.38	3.71–29.61	4.60–38.66	4.51–40.09
		20.84 ± 11.30	19.32 ± 10.59	23.26 ± 15.35	20.34 ± 13.80
	B	3.91–41.34	4.25–38.86	5.22–39.18	5.31–42.73
		29.79 ± 16.67	28.34 ± 15.59	28.96 ± 15.45	28.68 ± 15.97
Total Hardness (mg CaCO_3_/l)	S	1.39–6.44	1.39–7.77	1.48–8.41	1.74–8.99
		4.04 ± 2.10	4.72 ± 2.67	4.91 ± 2.83	5.00 ± 2.88
	B	2.31–8.41	2.54–8.47	2.47–8.87	2.50–8.90
		5.79 ± 2.69	6.48 ± 2.56	6.35 ± 2.99	5.50 ± 2.30
Total Alkalinity (g/l)	S	0.31–0.48	0.33–0.48	0.27–0.47	0.27–0.48
		0.38 ± 0.07	0.39 ± 0.05	0.36 ± 0.09	0.38 ± 0.08
	B	0.25–0.31	0.26–0.41	0.27–0.41	0.25–0.46
		0.26 ± 0.03	0.30 ± 0.06	0.31 ± 0.06	0.33 ± 0.09
Surfactant (mg LAS/l)	S	0.05–0.41	0.09–0.48	0.06–0.29	0.03–0.27
		0.16 ± 0.14	0.18 ± 0.11	0.15 ± 0.09	0.13 ± 0.07
	B	0.04–0.31	0.06–0.26	0.06–0.21	0.03–0.17
		0.13 ± 0.10	0.12 ± 0.08	0.11 ± 0.06	0.07 ± 0.06

*S* surface, *B* bottom

**Fig. 4:**
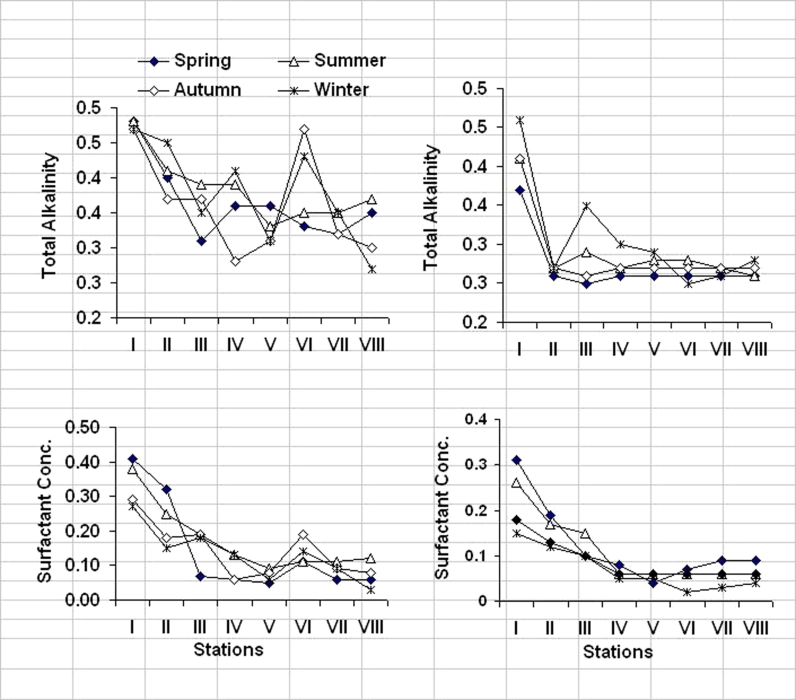
Regional and seasonal variations of total alkalinity (in grams per liter) and surfactant concentration (in milligrams LAS per liter) in El-Mex Bay Water (2008)

The variability of OOM content increased in the surface waters of El-Mex Bay from 1.8 to 5.2 times than those recorded in the bottom water. The regional distribution of OOM values were higher at the stations located at El-Umum Drain (station I, 6.38 mg O_2_/l) and in the vicinity of the drain (station III, 6.12 mg O_2_/l) and decreased further away of it at stations V, VII, and VIII. High significant inverse correlations were found between OOM values and salinity in the surface and bottom waters of El-Mex Bay (*r* = −0.81, *n* = 48, *p* < 0.002), and this confirmed that the organic matter is related to the allochthonous introduced to the bay from the surrounding area. The concentrations of organic matter in the surface and bottom waters of the bay were noticeably high in the warm seasons and low in the cold seasons (Fig. 4), and this is due to an increase in human population and activities during summer seasons.

#### Total alkalinity

Total alkalinity is simply expressed as the sum of equivalents of HCO_3_^−^, CO_3_^−2^, and hydroxides ions. In general, carbonate and bicarbonate ions are the main components contributing to the alkalinity in most natural waters. The distribution of total alkalinity (Fig. 4 and Table 1), during the period of study, showed a general decrease towards the northern region (stations VIII and VII, with surface water equal to 0.32 and 0.32 g CaCO_3_/l, respectively, and the bottom water equal to 0.27 g CaCO_3_/l) and north-western part of El-Umum Drain (station V, 0.33 and 0.27 g CaCO_3_/l for surface and bottom layers, respectively). Generally, the mean alkalinity values showed a high level in surface water compared to those in bottom water during the whole period of investigation (Fig. 4) except at stations V and II in August, in which the total alkalinity of surface water has slightly decreased than the bottom water. The high alkalinity of the surface water might result partially from the large amount of fresh water discharged into El-Mex Bay. This evidence can be supported by the strong negative correlation obtained between total alkalinity and salinity in the surface waters of the bay (*r* = −0.999, *n* = 48, *p* < 0.05). The pattern of distribution in the bottom water layer exhibits a narrow range of variation ranged between 0.27 g CaCO_3_/l at stations VIII, VI, V, and VII and 0.28 g CaCO_3_/l at stations IV and II.

The total alkalinity measurements in cold seasons decreased compared to that of warm seasons (Fig. 4). This can be supported by the relatively high positive correlations found between total alkalinity and water temperature of the surface and bottom layers of El-Mex Bay (*r* = 0.78, *n* = 48, *p* < 0.05) and reflect the important role of temperature in increasing photosynthetic activity in summer and decreasing it in winter and, consequently, causing the increase and decrease in total alkalinity, respectively.

#### Total hardness

Total hardness gives information about the concentration of Ca and Mg ions. Many of human activities and waste disposal in marine systems result in the leaching of chemicals which causes increase the level of total hardness. As shown in Fig. 3, low hardness values were recorded at El-Umum Drain (station I) and in the vicinity of the drain (stations II and III) and increases towards parts away from El-Umum Drain. Generally, the hardness values showed lower levels in surface water (ranged 4.55 ± 2.10 and 5.65 ± 2.83 mg/L as CaCO_3_), compared to those in bottom water (ranged 5.93 ± 2.30 and 6.95 ± 2.77 mg/L as CaCO_3_ during the whole period of investigation.

The low total hardness of the surface water might result from the large amounts of fresh water discharged from El-Umum Drain into the bay. This evidence can be supported by the strong positive correlation obtained between total hardness and salinity (*r* = 0.923, *n* = 48, *p* < 0.002). The Mediterranean Sea is characterized by calcareous substrata which induce strong mineralization of water and illustrate the higher levels of hardness for bottom layers compared to those in surface layers during the whole period of investigation. The seasonal variations in total hardness values were not evident.

### Anionic detergent (LAS) in El-Mex Bay region

#### Distribution of LAS concentrations in El-Mex Bay water

Linear alkylbenzene sulfonates have been found in wastewaters, seawaters, and sediment samples (Takada and Ishiwatari 1991), and these substances may be preserved in marine sediments indicated to the presence of domestic wastes (Raymundo and Preston 1992). Theoretically, the surfactants can be degraded and removed by chemical, physical, or biological processes in wastewater treatment plants (Ding et al. 1999). Large quantities of surfactant residues in wastewaters are directly discharged into the seawater.

The impact of surfactant pollution on the aquatic environment is very significant. The large-scale usage of surfactants and the increasing public concern over environmental issues have stimulated our interest to investigate their occurrence and behavior in the environment. The results of LAS concentrations in El-Mex Bay waters were undertaken to establish environmental concentrations and support seawater protection programs, and this monitoring can possibly help elucidate the behavior of these compounds and the effluents in the bay.

The regional and seasonal variations of anionic surfactant LAS at the surface and bottom water in El-Mex Bay are shown in Table 1 and graphically in Fig. 4. Station I was selected to check for El-Umum Drain contribution, as water enters the bay (Fig. 1). It was observed from these figures that stations I, III, and VI had the maximum LAS concentrations, the average values 0.24 ± 0.11 and 0.15 ± 0.06 mg LAS/l for stations I and II, respectively, and 0.14 ± 0.04 and 0.05 ± 0.02 mg LAS/l at station VI for surface and bottom layers, respectively. The high content is attributed to the presence of wastewater from station I and directly in front of station II and in front of El-Umum Drain (station VI) which discharges its wastewater into the coastal area. On the other hand, station VIII had the lowest LAS concentration at both surface 0.07 ± 0.04 mg LAS/l and bottom water 0.06 ± 0.02 mg LAS/l. The lower values of LAS concentration are due to the fact that station VIII was located away from El-Umum Drain at the entrances of the bay and away from land sources (offshore) besides being affected by the open seawater.

The spread of the wastewater in the bay was investigated by studying the distribution of LAS at surface and bottom layers during investigation period, and the results obtained seem to indicate a great variability in the concentration of LAS at all stations (Table 1 and Fig. 4) as a function of the season and distance from El-Umum Drain outlet. The concentration of surfactants dropped rapidly with distance from the point of discharge. The results also indicate that spreading of wastewater occurs almost exclusively in the brackish layer, while the underlying saline layer remains hardly affected. As can be seen, there is a strong reduction (down to 0.09–0.15 mg LAS/l) of LAS concentrations at stations III, VI, and IV. After a relatively long distance (stations VII and VIII), the concentration of LAS was dropped down below 0.08 mg LAS/l. The distribution of LAS residues in El-Mex Bay water column is characterized by pronounced maxima at the brackish water–seawater interface. The observed effect depends strongly on the wind conditions which can enhance the velocity and direction of surface currents as well as the mixing of brackish water and saline water. The brackish water–seawater interface is a very efficient barrier, which prevents spreading of surfactants into the deeper layers (Ahel and Terzic 2003).

High LAS concentration at stations I and III coincides with very low salinity 4.17 and 4.36 PSU, respectively, because these two stations are affected by drainage water discharged from El-Umum Drain through El-Mex pumping station. Such water is heavily contaminated with sewage and industrial wastes of Alexandria City. The amount of drainage water discharged into the sea fluctuates between 4.8 and 7.1 million m^3^/day with an annual average of about 2,420 million m^3^ (El-Sherif and Mahmoud 1991), so that the concentrations decrease significantly with distance from the point of discharge.

The biodegradation processes were shown to be seasonally dependent (George 2002). The seasonal average variations of LAS, in El-Mex Bay waters, are shown in Fig. 4. It is observed that the summer had the highest concentrations at both surface (0.13 mg LAS/l) and bottom (0.06 mg LAS/l) layers, due to increasing in water temperature in the summer season because temperature was found to have a decisive effect on the degradation rate. At the temperature representative of summer, the transformation of LAS is faster than under winter temperature conditions as reported in many works (Terzic et al. 1992; Perales et al. 1999; George 2002; Ahel and Terzic 2003). The highest concentration found in summer is attributed to an increase in population density and human activities in this area, which leads to an increase in domestic loading during this season. The volume of water discharged to El-Mex Bay through El-Umum Drain increases in summer seasons (Said et al. 1994).

In winter and autumn, LAS concentrations are the lowest at both surface and bottom layers (Fig. 4). In December and March, LAS concentrations at surface water are 0.12 and 0.11 mg LAS/l, respectively, which is relatively high when compared to those of bottom measurements (0.06 and 0.04 mg LAS/l, respectively). There is no clear relationship between LAS concentrations and water temperature. These data seem to show that continuous input of wastewater and surfactants occur in El-Mex Bay. It should be stressed that it is difficult to decouple the effect of biotransformation on LAS homologue composition from the changes induced by physicochemical characteristics, since the field observations on seasonally dependent transformation were confirmed by controlled laboratory experiments, which were conducted using natural microbial populations, originating from the same area of study (Jimenez et al. 1991). This seasonal variation is related to the amount of urban drainage and industrial waste that contains detergents.

The most characteristic feature of the surfactant distribution in marine environments is the distribution pattern on the vertical profile in water column. Generally, in most of the surface water samples, LAS concentrations were higher than in the bottom water at all stations (Fig. 4). This is due to the fact that the natural tendency of surfactants LAS is to accumulate first on the surface of the aqueous medium (air–seawater interface). Also, the degradation improves when it occurs at salinity value closest to the initial value of the water sediment (38.50–40.50 PSU); it can be attributed to the great concentration of bacteria as well as the physical–chemical characteristics of surfactants (Perales 1999). The same observations also have been discussed for the distribution of LAS in Abu-Qir Bay waters by Mahmoud (1991) and Eastern Harbor waters by Mahmoud (1989).

Correlation coefficient between LAS concentrations and salinity as a conservative parameter, showing the extent of mixing of seawater in the bay with sewage discharge, was calculated during investigation period of study. There is a significantly high negative correlation between them (−0.78, *n* = 48, *p* < 0.05). This inverse relationship confirms the occurrence of the sewage discharge containing detergents and indicating, thus, strong sedimentation or biodegradation pattern. The discharge affects not only the near shore stations but also reaches the offshore stations and stations away from the point of sewage discharge with low concentrations (stations VIII, V, and II) illustrated in Table 1 and represented graphically in Fig. 4. As for detergents, they are not degraded so fast during transportation as it affects the water offshore stations. It may also point to the possible use of detergents as tracers for pollution in the Mediterranean Sea. According to Stalmans et al. (1991), the decrease in LAS concentration with the distance from the source of contamination was faster than that predicted based on dilution only. It is anticipated that removal mechanisms from the seawater column include biodegradation (Rego et al. 1998), sorption to suspended solids (Prats et al. 1993), and precipitation with divalent cations (Xie et al. 1997). Since sorption of LAS on sediment is promoted when Ca^2+^ concentration increases (Prats 1993), adsorption is likely to be higher in estuaries where salinity gradients are steep (Temara et al. 2001). Since the vertical gradient of salinity found that there is not at all steep, the surfactant property of the compound must make a considerable contribution to causing the observed distribution. In fact, at greater distances from El-Umum Drain outlet, a certain vertical gradient is still found to be the main parameters that affect the distribution of LAS in the bay waters.

The relationship between LAS concentrations and the amount of dissolved oxygen in El-Mex Bay water was investigated. A linear regression analysis showed a highly negative relationship between the concentrations of LAS and dissolved oxygen, (*r* = −0.5, *n* = 48, *p* < 0.05) this may be attributed to high amount of dissolved oxygen that lead to an increase in the biodegradation rate of surfactants that were introduced to a natural system (Tunqay and Atun 1995). Therefore, introduction of biodegradable materials lead to depletion of the dissolved oxygen in water. In some cases, this deficit of oxygen cannot be overcome by reaeration at the air–water interface because of foaming, thus the diminished level of dissolved oxygen may be so low that higher forms of aquatic life cannot survive. These agree with the data collected at stations I and II where municipal wastewater was dumped directly without any treatment through El-Umum Drain. High LAS concentration at stations I and III is coincided with very low amounts of dissolved oxygen 1.87 ± 1.3 and 1.31 ± 0.6 mg O_2_/l, respectively. The oxidizing conditions and high amounts of dissolved oxygen, at the rest of stations, encouraged bacterial action on a large number of organic compounds easily biodegradable in such conditions, and LAS concentrations will be diminished. The concentration of LAS at surface water was higher than that in the bottom water at all stations and seasons. This was not only due to the tendency of aromatic surfactants to accumulate at the phase boundaries which are in agreement with their amphiphilic nature (González-Mazo and Gόmez-Parra 1996) but also due to the rate of biodegradation that was remarkably accelerated in the presence of sediment and relatively low bottom water salinity.

Comparing the levels of LAS found in the study area water with those reported in previous studies in other regions of Mediterranean Sea, Red Sea, and Nile Delta, it is observed that the detergents content in the Red Sea coastal water in front of Hurgada was 0.1–0.26 mg LAS/l (Mahmoud 1998), and it is remarkably less than that recorded by Mahmoud (1989) in the Eastern Harbor (0.05–3.06 mg LAS/l). The present data are less than that recorded by El-Sherif and Mahmoud (1991) and by Mahmoud (1991) in El-Mex Bay (0.08–1.70 mg LAS/l) and Abu-Qir Bay and (ND-4.1 mg LAS/l) respectively. The results are similar to the brackish water of Lake Borollos in the Nile Delta (0.17 mg LAS/l) (Beltagy and Mahmoud 1988) and higher than those recorded at El-Agamy (0.02–0.08 mg LAS/l) (Mourad and Abd-Allah 1995). Maryout Lake represents the highest toxic area in Alexandria City (3.6–18.6 mg LAS/l) (Mourad and Abd-Allah 1995).This high level of toxicity is attributed to the huge amount of industrial and sewage discharge into these areas. El-Sherif and Mahmoud (1991) reported that the anionic detergents concentration had negative effects on the standing crop of phytoplankton in El-Mex Bay. The seasonal variations of El-Mex Bay surface water showed an outstanding peak of phytoplankton during spring, and it consisted mostly of green algae accompanied by a low concentration of detergents.

#### Ca (LAS)_2_ precipitation boundary diagram

In the receiving water, however, there are three basic mechanisms playing an important role in the interactions between multiple present components, decreasing LAS concentrations as well as the other substances (Verge et al. 1997, 2000). Such mechanisms are biodegradation, adsorption, and precipitation. With very few exceptions, these mechanisms are responsible for surfactant toxicity observations in the laboratory usually being higher than those in real environmental samples (Quiroga et al. 1989). Any of these mechanisms will modify the bioavailability of a given substances and, consequently, its ecotoxicological properties in the water column. LAS biodegradation, precipitation, and adsorption in suspended organic matter or sediments enhance their elimination from marine environment and consequently reduce their effects on aquatic organisms.

Depending on Ca^2+^ concentration (water hardness), LAS can be solubilized or precipitated, in this case, LAS precipitation as Ca (LAS)_2_ from the water column, and there exists a decrease of the bioavailable fraction for aquatic organisms and, consequently, a toxicity reduction. A tentative comparison between LAS concentrations and calcium ion concentrations (water hardness) during investigation period of study was calculated. Water hardness also can be used as an indicator variable, showing the mixing between brackish water and seawater in El-Mex Bay. There was a significantly high negative correlation between LAS contents and calcium ions concentrations (*r* = −0.67, *n* = 48, *p* < 0.05), and this indicating, thus, strong precipitation of LAS as Ca(LAS)_2_ when Ca^2+^ concentration increases and seems to promote cooperative sorption at high surfactant and calcium ion concentrations (Garcĺa et al. 2002). Calcium ion enhances adsorption in bilayer and would confirm the affinity for the anionic surfactant after an initial adsorption in a monolayer. Thus, LAS sorption on sludge and sediment particles can be facilitated in hard water owing to the decrease of the electrostatic repulsion ionic head of LAS. Furthermore, calcium ions could adsorb directly to the sludge particle, yielding positively charged sites onto which negatively charged LAS homologues can adsorb.

**Fig. 5:**
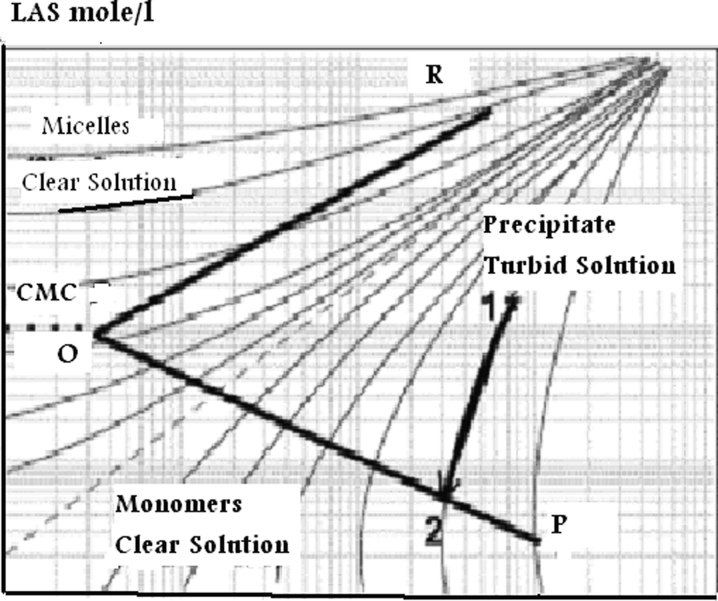
Ca (LAS)_2_ typical precipitation boundary diagram Verge et al. (2000)

Verge et al. (1997, 2000) introduced the effect of LAS precipitation, due to water hardness, on bioavailability and consequently on toxicity. The biological test used was the acute toxicity to *Daphnia*. The basic tool for their study has been the Ca(LAS)_2_ precipitation boundary diagram which is based on the determination of the turbidity of various solutions at different LAS and Ca concentrations. Calcium precipitation boundary diagrams are very useful tools to evaluate anionic surfactants–calcium ion interactions including an estimation of critical micelle concentration (CMC), solubility product, as well as information concerning the interactions of calcium ions with micelles in solution. These diagrams are essentially phase diagrams for anionic surfactant (LAS) as a function of Ca^2+^ concentration. They are drawn on a log–log plot in which the precipitation boundary represents the onset precipitation observed. The diagram is divided into regions representing areas in which there are only monomers (zone I, transparent), monomers + micelles (zone II, turbid), or only micelles (zone III, transparent). Moving vertically on the graph, the surfactant concentration increases, and while moving horizontally, the Ca^2+^ concentration increases. Application of the data concerning LAS and Ca^2+^ concentrations in log form during the investigation period of study to the basic boundary diagram of LAS (Fig. 5), which have been drawn by Verge et al. (1997, 2000), was characterized by three different zones.

##### Zone I or monomer zone

Below the OP line is corresponding to LAS only in monomer form. LAS and Ca^2+^ concentrations data for stations I and III were located in this zone. It is a transparent zone because the solubility product of Ca (LAS)_2_ has not been reached yet. The boundary OP represents the points corresponding to LAS and Ca^2+^ concentration and matching the general equation:

$$ {\left( {LA{S^{ - }}} \right)^2} \times \left( {C{a^{{2 + }}}} \right) = {K_{{sp}}} $$

where (LAS^−^) is the linear alkylbenzene sulfonates concentration in mole per liter; (Ca^2+^) is the calcium ion concentration in mole per liter; *K*_sp_ is the solubility product of Ca (LAS)_2_. Point O corresponds approximately to CMC, above where there are monomers and micelles exist.

##### Zone II or micellar zone

This lies above the OR line. In this zone, there are sufficient LAS^−^ micelles to sequester the Ca^2+^ concentration present, and consequently, the solution is transparent.

##### Zone III or precipitation zone

This is the area between lines OR and OP, and data obtained from El-Mex Bay from stations VII to VIII lie in this zone, where all solutions are turbid (monomers and micelles). This illustrates that, when surfactants are dumped in the seawater, the high ionic strength of the medium causes a fall in their CMC, and consequently, their solubility is greatly reduced (Quiroga et al. 1989). As a result, these materials accumulate in the sediments close to waste outlet. The thin curve lines in Fig. 5 represent the evolution of a given point within the precipitation zone until reaching the equilibrium (OP or OR lines), corresponding to a saturated solution. For instance, Ca^++^ and LAS^−^ concentrations of point 1 (within the precipitation zone) will commence the precipitation of Ca (LAS)_2_, and once the equilibrium is reached, the composition of final saturated solution will correspond to point 2.
